# Exploring the in vitro stability of insulin degrading enzyme as a potential biomarker for neurocognitive disorders and Alzheimer's disease risk

**DOI:** 10.1016/j.plabm.2024.e00400

**Published:** 2024-05-16

**Authors:** Helena Kullenberg, Marie M. Svedberg

**Affiliations:** Department of Health Promoting Science, Sophiahemmet University, Stockholm, Sweden

**Keywords:** Alzheimer's disease, Diabetes type 2, Insulin-degrading enzyme, In vitro stability, Metabolic function, Storage conditions

## Abstract

Insulin degrading enzyme (IDE) plays a critical role in degrading insulin and beta-forming proteins, implicating its significance as a biomarker in metabolic dysfunction and neurocognitive disorders, including Alzheimer's disease (AD). Understanding the impact of pre-analytic conditions of in vitro IDE levels is imperative for reliable biomarker assessment. This study explored the influence of freeze-thaw cycles, storage temperature, and storage time on IDE levels in human serum.

Serum samples from seven healthy volunteers were subjected to various storage conditions, including refrigeration (4 °C) and freezing (−20 °C and −80 °C) for 24 h and six months, with differing freeze-thaw cycles. In vitro IDE levels were measured at 24 h and after 6 months using ELISA.

Results indicate that while short-term storage at either −20 °C or −80 °C yielded similar IDE levels, prolonged storage and multiple freeze-thaw cycles significantly impacted IDE stability, with colder temperatures exhibiting better preservation.

Although further research with larger cohorts and longer storage time is warranted to establish clinical significance, our study suggests preferential use of unthawed samples or consistent freeze-thaw conditions for accurate IDE assessment. Thus, optimizing sample storage conditions is paramount for reliable IDE biomarker analysis in clinical and research settings.

## 1 Introduction

Insulin degrading enzyme (IDE) is a zinc-metalloprotease found in tissue and extracellular fluids [1]. The enzyme is vital for degrading insulin and beta-forming proteins such as beta-amyloid. Therefore, it has been suggested as a link between metabolic dysfunction and neurocognitive disorders [2]. It has also been suggested as a potential biomarker to identify individuals at risk of accumulating extraneuronal beta-amyloid and consequently developing Alzheimer's disease (AD) [3].

We have earlier demonstrated higher levels of IDE in serum from individuals with type 2 diabetes compared to healthy controls [4,5] and suggested a correlation between IDE levels and inflammation in individuals with AD [6]. In this work, we have not found any information on the stability of IDE and how the biomarker would be affected by handling and storage conditions.

Understanding how the biomarker of interest is affected by pre-analytic conditions is crucial in both clinical practice and research. Pre-analytic parameters, such as sampling and storage, account for about three-quarters of the errors that occur throughout the analytical process [7].

It is generally recommended to avoid repeating freeze and thaw cycles by manufacturers of analytic material, but in a research setting, this can be challenging to comply with. Recruitment of participants, and hence participant samples, can be time-consuming, and it is usually unavoidable to freeze samples until a sufficient number has been obtained. Additionally, conducting all analyses for a study in a single thaw is rarely feasible, resulting in multiple freeze-thaw cycles [8]. Therefore, understanding the optimal conditions for storing samples and planning for analyses is crucial. In addition, understanding which samples are suitable for use and which should be excluded is essential. However, analyzing in vitro levels of IDE as a potential biomarker involves an investigation of its freeze-thaw and temperature stability.

## 2 Aim

The aim of this study was to explore the impact of freeze-thaw cycles and storage temperature on in vitro levels of IDE in human serum.

## 3 Materials and methods

### 3.1 Participants

Healthy volunteers were recruited from Sophiahemmet Hospital in Stockholm, Sweden. Inclusion criteria comprised individuals aged over 18 with no chronic health conditions or ongoing drug treatment. Samples from a total of 7 subjects were included in this study.

All participants provided their informed consent to participate, and the study was conducted in accordance with the Declaration of Helsinki regulation [9]. The Swedish Ethical Review Authority approved the study (Dnr. 2022-02633-02).

### 3.2 Sample collection

Non-fasting blood samples were drawn from an antecubital vein in two anticoagulant-free tubes (6 ml) and one EDTA tube (2 ml). EDTA blood was used immediately for analyses with point-of-care platforms. Anticoagulant tubes were left to coagulate for 30 min and then centrifuged for 15 min at 2000 rpm. The serum was aliquoted and prepared as described below.

### 3.3 Preparation of study samples

To assess stability depending on the storage conditions, we stored serum samples in various setups, including refrigeration (4 °C) for 24 h, freezing at the time of blood collection (−20 °C and −80 °C, respectively), for 24 h and six months, with a one, three and ten thaw cycles, respectively.

The first analysis was conducted 24 h after blood collection. In this analysis, we included refrigerated samples, samples frozen at −20 °C or −80 °C degrees and thawed once, and samples frozen at −20 °C or −80 °C and thawed three times. Subsequently, we conducted a re-analysis of the serum samples after six months. At the six-month time point, we utilized serum samples frozen at the time of blood collection to −20 °C or −80 °C, stored for six months, and thawed once for analysis, and serum frozen at the time of blood collection (at −20 °C and −80 °C) underwent ten thawing cycles over the six-month period before analysis.

Freeze-thaw cycles were created by thawing solid frozen samples for 1 h at room temperature and refreezing them for at least 8 h before repeating the cycle.

### 3.4 Biochemical analysis

IDE levels were measured using a human IDE ELISA kit (Human Insulin-degrading enzyme ELISA Kit, AH Diagnostics), with the minimum detection concentration at 0.037 ng/ml. Coefficients of variation (CV) for all assays were below 10 %.

Blood glucose was measured with HemoCue® 301, and glycated hemoglobin A1c (HbA1c) was measured with AfinionTM 2 (Abbott Rapid Diagnostics, Maidenhead, UK).

All samples, reagents, and buffers were prepared according to the manufacturer's instructions. We used an automated HydroWash from Tecan for all required wash steps. All samples were analyzed anonymously.

### 3.5 Statistical analysis

Due to the small sample size, descriptive data were presented as median and interquartile range. Differences between time points were analyzed using Friedman's two-way analysis of variance by rank test and differences between storage temperature using the Wilcoxon Signed Rank test. All tests were two-tailed, and a p-value less than 0.05 was considered statistically significant. Statistical analysis was performed with SPSS 27.0, and figures were produced in Prisma GraphPad v9.

## 4 Results

### 4.1 Study sample

We included 7 volunteers, 3 men and 4 women - aged between 29 and 78 years (median 48, interquartile range 41–78). None of the participants were diagnosed with a metabolic condition, had abnormal blood glucose (median 5.0 mmol/l, interquartile range 4.7–5.3), elevated HbA1c (median 35 mmol/mol, interquartile range 33–37), or BMI above 30 (median 24.7 kg/m2, interquartile range 23.2–26.7).

### 4.2 Freeze-thaw cycles

Analysis of samples exposed to none, one, three, or ten freeze-thaw cycles demonstrated no significant differences for samples stored at −80 °C regardless of the number of cycles (Table 1). However, for samples stored at −20 °C, significant differences were observed between samples refrozen ten times and samples thawed once or thrice (Table 1). Notably, there was no statistical difference between IDE levels in fresh samples and those frozen at −20 °C and thawed ten times (Table 1).

**Table 1:** In vitro levels of serum insulin-degrading enzyme (ng/ml) depending on storage conditions.

		Storage temperature		
		−20 °C	−80 °C	*Sig*.^a^
Number of freeze-thaw cycles	0	5.19 (4.38–10.34)	5.19 (4.38–10.34)	
	1	4.54 (3.58–7.55)	4.79 (3.12–5.68)	1.000
	3	4.58 (3.55–10.01)	4.50 (3.07–6.85)	0.063
	10	6.94 (6.49–17.58)	6.60 (3.91–9.06)	**0.028**
	*Sig*.^b^	**0.010**^c^	0.134	
Storage time	Refrigerated 24 h	5.19 (4.38–10.34)	5.19 (4.38–10.34)	
	Frozen 24 h	4.54 (3.58–7.55)	4.79 (3.12–5.68)	1.000
	Frozen 6 months	5.60 (4.02–14.61)	4.84 (4.30–8.15)	0.063
	*Sig.*^d^	0.102	0.368	

a Statistical significance of the difference between storage in −20 °C or −80 °C. Difference analyzed with Wilcoxon signed rank test.

b Statistical significance of difference depending on the number of freeze-thaw cycles. Difference analyzed with Friedman's two-way analysis of variance by rank test.

c There was a statistically significant difference between 1 or 10 freeze-thaw cycles (*p* = 0.011) and 3 or 10 freeze-thaw cycles (*p* = 0.043) after correction with Bonferroni.

d Statistical significance of difference depending on storage time. Difference analyzed with Friedman's two-way analysis of variance by rank test.

### 4.3 Storage temperature

When we compared samples frozen and thawed the same number of times but stored at different temperatures, we found no difference between samples frozen overnight and thawed once (Table 1). Nonetheless, there was a significant difference between samples frozen and thawed 10 times, depending on the freezing temperature. In addition, there was a difference between samples stored for 6 months and samples thawed three times, but it was only borderline significant (Table 1).

### 4.4 Storage time

**Fig. 1:**
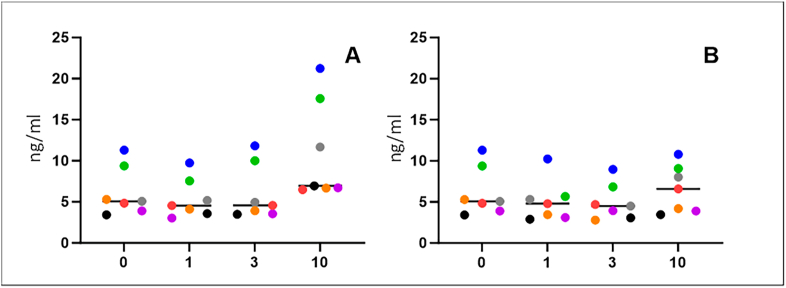
In vitro levels of serum insulin-degrading enzyme (ng/ml). Analysis of individual samples exposed to various conditions. 0; samples refrigerated for 24 h, 1; samples frozen and thawed once, 3; frozen and thawed three times, and 10; frozen and thawed ten times. Freeze-thaw cycles were performed at −20 °C (A) or −80 °C (B).

No statistically significant differences were found when analyzing samples stored at 4 °C compared to samples stored frozen for 24 h or six months. (Table 1). However, the difference between IDE levels in each sample was less prominent in samples stored at −80 °C (Fig. 1).

## 5 Discussion

In this study, we investigated how serum levels of IDE were affected by storage temperature and the number of freeze-thaw cycles. We could demonstrate that storage temperature and freeze-thaw cycles may influence serum IDE levels in human blood.

Many biomarkers are affected by pre-analytical handling, and it is, therefore, essential to be aware of this when collecting samples and planning analyses. This is very important in a clinical setting where medical decisions are based on laboratory analysis. Still, it is also essential in research as different pre-analytical handling may mean that results from various studies cannot be compared [7].

Here, we could show that when samples were frozen overnight, the freezing temperature had no significance, as there was no difference between samples frozen at −20 °C or −80 °C. This suggests that for short-term storage, the temperature is less critical. The assay instructions suggest that samples can be stored in the refrigerator for up to 5 days before analysis, a guideline that may be correct. However, the differences became slightly more extensive when the samples were subjected to three freezing cycles. At ten freeze-thaw cycles, the difference became significant—even with a storage duration of 6 months, a difference was observed between samples stored at −20 °C and −80 °C, which, although it did not reach statistical significance, approached a level of significance. It is worth noting that samples stored at −80 °C showed more minor individual differences and a smaller spread than those held at −20 °C, indicating that colder temperatures are preferable for longer than temporary storage.

The difference in IDE levels between fresh samples, samples frozen for 24 h, or samples frozen for six months was not statistically significant. Still, in general, the serum levels of IDE decreased in the frozen samples compared to samples analyzed after being stored in a refrigerator (4 °C). Although not statistically significant, the trend was clear for the analyzed samples, especially those frozen to −80 °C, and may need to be considered. Therefore, it is not advisable to mix fresh samples with those that have been frozen, regardless of the freezing temperature of the sample.

However, six months is now a relatively short time to store samples. In AD research, biobank samples are often used, and the time between sample collection and analysis can be extensive [6]. This study has examined serum, but plausibly, IDE is similarly affected in other types of samples, such as brain tissue. Thus, further studies must investigate whether IDE levels change over time, also in samples stored at −80 °C. Overall, it seems essential to know how samples have been handled and that it has been handled in a reproducible way.

Moreover, as measuring IDE levels is a novel biomarker approach, there is a lack of prior research for comparison, and a defined cut-off or reference interval is not yet established. Consequently, evaluating whether the differences arising from storage conditions are clinically significant remains challenging. A study with a larger sample size would likely be necessary to explore this aspect further.

## 6 Conclusion

In this study, serum IDE levels were affected by storage conditions. It might not have to be considered if samples are used within a short period, but if samples are stored over time, −80 °C is preferred. It would be recommended to use unthawed samples in research studies investigating levels of IDE. Still, if samples must be frozen and thawed repeatedly, using samples exposed to the same number of cycles and the same storage temperature is essential.

## Funding

This study was funded by Stohnes Foundation.

## Institutional review board statement

The study was conducted in accordance with the Declaration of Helsinki regulation and approved by the Swedish Ethical Review Authority (Dnr. 2022-02633-02).

## Informed consent statementstatement

All participants provided their written informed consent to participate.

## CRediT authorship contribution statement

**Helena Kullenberg:** Writing – review & editing, Writing – original draft, Visualization, Validation, Software, Investigation, Formal analysis, Data curation, Conceptualization. **Marie M. Svedberg:** Writing – review & editing, Validation, Supervision, Resources, Project administration, Methodology, Investigation, Funding acquisition, Conceptualization.

## Declaration of competing interest

The authors declare no conflict of interest.

## Data Availability

Data will be made available on request.

## References

[1] Kurochkin I.V., Guarnera E., Berezovsky I.N. (2018). Insulin-degrading enzyme in the fight against Alzheimer's disease. Trends Pharmacol. Sci..

[2] de la Monte S.M. (2014). Type 3 diabetes is sporadic Alzheimers disease: mini-review. Eur. Neuropsychopharmacol.

[3] Sun J., Xia W., Cai R., Wang P., Huang R., Sun H. (2016). Serum insulin degrading enzyme level and other factors in type 2 diabetic patients with mild cognitive impairment. Curr. Alzheimer Res..

[4] Kullenberg H., Rossen J., Johansson U.B., Hagströmer M., Nyström T., Kumlin M., Svedberg M.M. (2022;Sep). Increased levels of insulin-degrading enzyme in patients with type 2 diabetes mellitus. Endocrine.

[5] Kullenberg H., Rossen J., Johansson U.B., Hagströmer M., Nyström T., Kumlin M., Svedberg M.M. (2024 May). Correlations between insulin-degrading enzyme and metabolic markers in patients diagnosed with type 2 diabetes, Alzheimer’s disease, and healthy controls: a comparative study. Endocrine.

[6] Kullenberg H., Nyström T., Kumlin M., Svedberg M.M. (2023 Jul 5). Correlation between insulin-degrading enzyme versus total tau and selected cytokines in patients with Alzheimer's disease compared to non-demented controls. Neuroendocrinol. Lett..

[7] Plebani M. (2012). Quality indicators to detect pre-analytical errors in laboratory testing. Clin. Biochem. Rev..

[8] Bai M., Liu Z.L., Zhou Y.Y., Xu Q.X., Liu T.X., Tian H.G. (2023 Jan). Influence of diverse storage conditions of double-stranded RNA in vitro on the RNA interference efficiency in vivo insect Tribolium castaneum. Pest Manag. Sci..

[9] World Medical Association (2013). WMA Declaration of Helsinki: Ethical principles for medical research involving human subjects. https://www.wma.net/what-we-do/medical-ethics/declaration-of-helsinki/.

